# miR-299-3p suppresses cell progression and induces apoptosis by downregulating PAX3 in gastric cancer

**DOI:** 10.1515/biol-2021-0022

**Published:** 2021-03-23

**Authors:** Zhenfen Wang, Qing Liu, Ping Huang, Guohao Cai

**Affiliations:** Department of Gastrointestinal Surgery, Hainan General Hospital, No. 19 Xiuhua Rd, Xiuying District, 570311, Haikou, Hainan, China

**Keywords:** miR-299-3p, PAX3, gastric cancer, GC biomarkers, GC therapy

## Abstract

Gastric cancer (GC) is ranked the fourth leading cause of cancer-related death, with an over 75% mortality rate worldwide. In recent years, miR-299-3p has been identified as a biomarker in multiple cancers, such as acute promyelocytic leukemia, thyroid cancer, and lung cancer. However, the regulatory mechanism of miR-299-3p in GC cell progression is still largely unclear. Cell viability and apoptosis tests were performed by CCK8 and flow cytometry assay, respectively. Transwell assay was recruited to examine cell invasion ability. The interaction between miR-299-3p and PAX3 was determined by the luciferase reporter system. PAX3 protein level was evaluated by western blot assay. The expression of miR-299-3p was downregulated in GC tissues and cell lines (MKN-45, AGS, and MGC-803) compared with the normal tissues and cells. Besides, overexpression of miR-299-3p significantly suppressed proliferation and invasion and promoted apoptosis in GC. Next, we clarified that PAX3 expression was regulated by miR-299-3p using a luciferase reporter system, qRT-PCR, and western blot assay. Additionally, downregulation of PAX3 repressed GC cell progression. The rescue experiments indicated that restoration of PAX3 inversed miR-299-3p-mediated inhibition on cell proliferation and invasion. miR-299-3p suppresses cell proliferation and invasion as well as induces apoptosis by regulating PAX3 expression in GC, representing desirable biomarkers for GC diagnosis and therapy.

## 1 Introduction

Gastric cancer (GC) has developed into the fourth leading cause of cancer-related deaths with more than 75% mortality rate worldwide [1,2]. Generally, GC is induced by poor dietary habits, *Helicobacter pylori* infection, gastric mucosal inflammation, and atrophy [3,4]. Advanced diagnostic tools and medical examinations have facilitated early detection of GC. However, most patients are diagnosed at an advanced stage, which vitiated the treatment outcomes, leading to low 5-year survival rate close to 27% [5,6,7]. Therefore, exploration of the pathogenesis of GC is essential for the development of novel therapy strategies.

MicroRNAs (miRNAs) refer to a class of small noncoding RNAs comprising 18–23 endogenous oligonucleotides [8]. They specifically participate in tumorigenesis, metabolism, proliferation, differentiation, epithelial-to-mesenchymal transition (EMT), and metastasis by base-pairing their messenger RNA (mRNA) and resulting in posttranscriptional gene regulation, mRNA degradation, and protein translation suppression [9,10,11]. Thus, the differential expression of miRNA has been observed in various cancers [12,13]. For instance, the abundance of miR-299-3p markedly accelerated cell growth and G1/S transition in acute promyelocytic leukemia through targeting p21Cip1/Waf1 [14]. On the contrary, miR-299-3p showed a low level of expression in thyroid cancer, and upregulation of miR-299-3p significantly hindered cell progression *in vitro* and *in vivo* by regulating SHOC2 expression [15]. Interestingly, miR-299-3p promoted chemosensitivity to doxorubicin by directly targeting ATP binding cassette E1 in lung cancer [16]. However, the biological role of miR-299-3p in GC cell growth remains unknown.

Paired box 3 (PAX3), an essential member of the paired box family gene, is a highly conservative transcriptional factor located at the 35–37 region of the chromosome 2 long arm [17,18]. Typically, PAX3 is involved in tissue development during the embryonic stage and the maintenance of stem cell niches by inhibiting b-Tubulin-III expression [19]. More importantly, PAX3 influences cell self-renewal, migration, and differentiation orientation alteration through multiple pathways [20]. For instance, PAX3 was reported to accelerate human glioma cell proliferation through regulating WNT/β-Catenin signaling pathways [21]. PAX3/FOXO1 fusion accelerated PAX3/FOXO1-positive alveolar rhabdomyosarcoma aggregation by regulating PPP2R1A [22]. Conversely, Wei Liu et al. considered that PAX3 served as a tumor suppressor in thyroid cancer by regulating transcription factor FOXO3a [23]. A recent study shows that the expression of PAX3 might be associated with the prognosis of GC [24]. Another research published in GUT indicated that PAX3 binding to NOC3L affects GC cell growth [25]. These results demonstrate that PAX3 might play a key role in GC progression. However, the precise mechanism of PAX3 in GC is not known. Thus, an investigation of the function of PAX3 in GC is necessary.

In the present study, we explored the function of miR-299-3p during GC cell progression. Examination of miR-299-3p expression by qRT-PCR showed that miR-299-3p was downregulated in GC tumors and cell lines, suggesting the suppressive role of miR-299-3p. Moreover, we demonstrated that PAX3 is a target of miR-299-3p. Besides, miR-299-3p regulates cell progression by targeting PAX3. Our research provides promising targets for GC treatment.

## 2 Materials and methods

### 2.1 Patient tissues

Fresh GC tumor tissues and the corresponding normal tissues were collected from 48 GC patients who underwent surgery in Hainan General Hospital. Then, the tissues were transferred and stored at −80°C immediately until use. All the participants have not received preoperative treatment before surgery.


**Informed consent:** Informed consent has been obtained from all individuals included in this study.
**Ethical approval:** The research related to human use has been complied with all the relevant national regulations, institutional policies, and in accordance with the tenets of the Helsinki Declaration and has been approved by the Ethics Committee of Hainan General Hospital.

### 2.2 Cell culture

GC cell lines MKN-45, AGS, and MGC-803 and human gastric mucosal epithelial cell line GES-1 were purchased from Cell Bank of Chinese Academy of Sciences (Shanghai, China). All the cells were maintained in DMEM (Gibco, Grand Island, NY, USA) supplemented with 10% FBS and 0.05% penicillin/streptomycin (Invitrogen, CA, USA) at 37°C in a 5% CO_2_ incubator.

### 2.3 Cell transfection

The miR-299-3p mimics and miRNA negative control (miR-NC) were purchased from RIBOBIO (Guangzhou, China). Small interfering RNA (siRNA) targeting PAX3 (si-PAX3), siRNA negative control (si-NC), and PAX3 were synthesized by Genepharma (Shanghai, China). These plasmids were transfected in MKN-45 and AGS cells using Lipofectamine 2000 (Invitrogen).

### 2.4 Quantitative reverse transcription polymerase chain reaction (qRT-PCR)

Total RNA was isolated from GC tissues and cells using TRIzol reagent (Invitrogen) by following manufacturers’ instructions. The cDNA for miR-299-3p and PAX3 was synthesized using RNA by All-in-One™ First-Strand cDNA Synthesis Kit (FulenGen, Guangzhou, China). qRT-PCR was performed using SYBR green (Applied Biosystems, Foster City, CA, USA) according to the standard procedure. The primers for miR-299-3p and PAX3 were as follows: miR-299-3p (Forward, 5′-TTCAGTGTAAACATCCTCGACTG-3′; Reverse, 5′-TGGCAATGTCGTGGAGTCG-3′); PAX3 (Forward, 5′-GCTGGGAAATCCGAGACA-3′; Reverse, 5′-CCTCCTCCTCTTCACCTTT-3′).

### 2.5 CCK8 assay

CCK8 assay was used to evaluate the cell proliferation ability of GC cells. Briefly, transfected MKN-45 and AGS cells (5,000 cells/well) were seeded onto 96-well plates. After continuous incubation for 24, 48, and 72 h at 37°C in 5% CO_2_ incubator, 10 μL of CCK8 reagent (Beyotime, Shanghai, China) was added to each well for another 2 h. The optical density (OD) value at 450 nm was read by a spectrophotometer (Thermo Fisher Scientific, Waltham, MA, USA).

### 2.6 Flow cytometric analysis

Transfected MKN-45 and AGS cells were seeded on a 24-well plate and continuously incubated for 48 h. The cells were then collected and stained using Annexin V-FITC/PI Apoptosis Detection Kit (Vazyme, Nanjing, China) for 20 min. The apoptotic rate was counted by BD FACS Canto II (BD Biosciences, Franklin Lakes, NJ, USA) flow cytometry.

### 2.7 Transwell assay

Cell invasion ability was examined by transwell assay. The upper chamber was coated with Matrigel (Becton Dickinson, Franklin Lakes, NJ, USA) for 4 h. Then, transfected MKN-45 and AGS cells were seeded on the upper chamber coated with Matrigel and continuously incubated for 48 h. Afterward, noninvasive cells were removed from the upper chamber using a PBS-soaked cotton swab. The invasive cells were fixed with 4% paraformaldehyde and stained with 0.1% crystal violet for 10 min, respectively. The visible cells were counted manually under the microscope.

### 2.8 Dual-luciferase reporter assay

PAX3 sequences harboring wild-type or mutant-type miR-299-3p binding sites were amplified and inserted into the downstream of the stop codon of psiCHECK2 dual-luciferase reporter plasmids (Cat. no C8021; Promega, Madison, WI, USA) and named as PAX3-Wt or PAX3-Mut (Geneseed, Guangzhou, China). Then the wild-type and mutant-type luciferase vectors (PAX3-Wt and PAX3-Mut) were co-transfected with miR-299-3p or miR-NC into MKN-45 and AGS cells using Lipofectamine 2000 transfection reagent. Luciferase activities were evaluated by dual-luciferase assay system (Promega, Madison, WI, USA).

### 2.9 AGS xenograft model

1 × 10^6^ AGS cells stable transfected with miR-299-3p were injected into athymic BALB/C mouse (6 weeks old) purchased from Beijing Vital River Laboratory Animal Technology Co., Ltd. (Beijing, China). Then, tumor volume (length × width × width/2) was measured every 5 days. Twenty-eight days later, the mice were sacrificed, and the tumor weight was analyzed. Tumor tissues are snap frozen at −80° for gene expression analysis.


**Ethical approval:** The research related to animal use has been complied with all the relevant national regulations and institutional policies for the care and use of animals.

### 2.10 Statistical analysis

All the experiments were conducted at least three times, and data were presented as mean ± standard deviation (SD). Statistical analysis was carried out using SPSS 13.0 software (Chicago, IL, USA) and GraphPad Prism 7 (GraphPad Inc., San Diego, CA, USA). A *p*-value of <0.05 was considered statistically significant.

## 3 Results

### 3.1 miR-299-3p is downregulated in GC tumor tissues and cell lines

**Figure 1:**
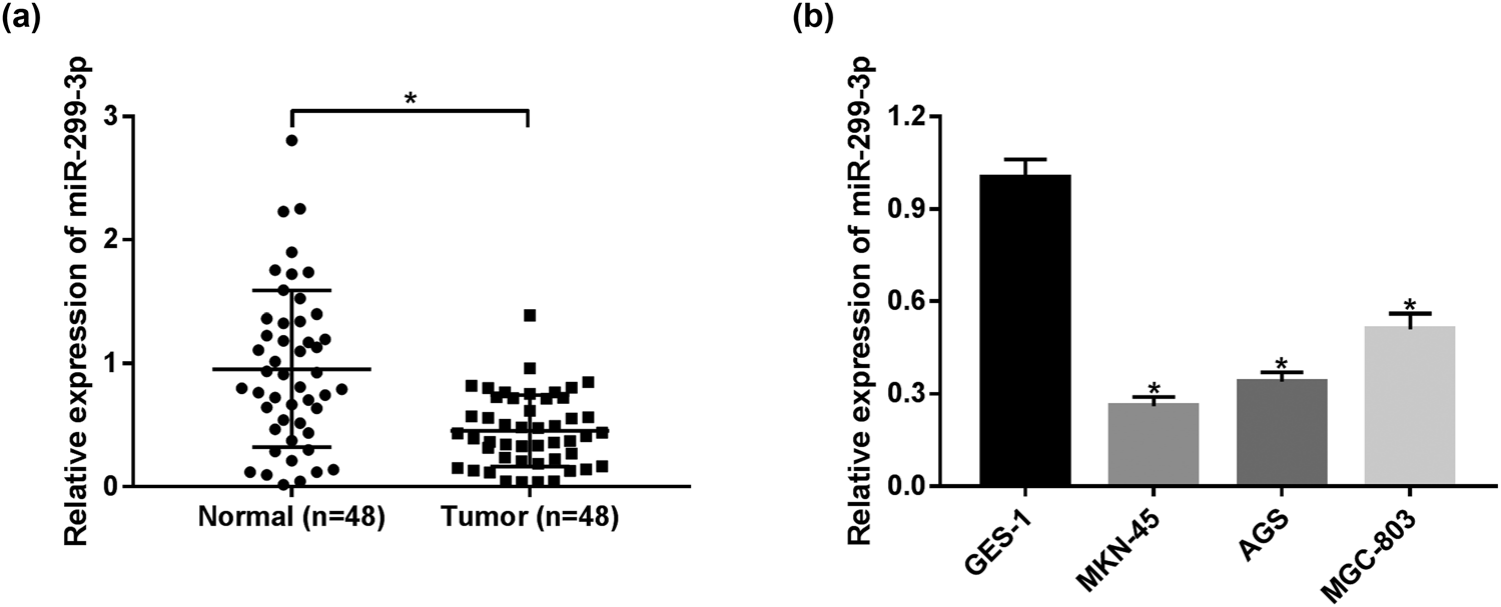
The expression of miR-299-3p in GC tumors and cell lines. (a) The expression of miR-299-3p in GC tumor tissues compared with the corresponding adjacent normal tissues measured by qRT-PCR. (b) The expression of miR-299-3p in GC cell lines (MKN-45, AGS, and BGC-823) compared with human gastric mucosal epithelial cell line GES-1. *P < 0.05.

The relative expression of miR-299-3p in 48 pairs of GC tumors and normal tissues was measured by qRT-PCR. As illustrated in Figure 1a, there is a variable 2 to 4-fold difference in miR-299-3p expression in GC tumors compared with the corresponding normal tissues. Consistently, miR-299-3p expression was relatively lower in GC cell lines (MKN-45, AGS, and MGC-803) compared with human gastric mucosal epithelial cell line GES-1 (Figure 1b). From these data, it is speculated that miR-299-3p might play a role of the tumor inhibitor in GC progression.

### 3.2 Overexpression of miR-299-3p suppresses cell proliferation and invasion and promotes apoptosis in GC

**Figure 2:**
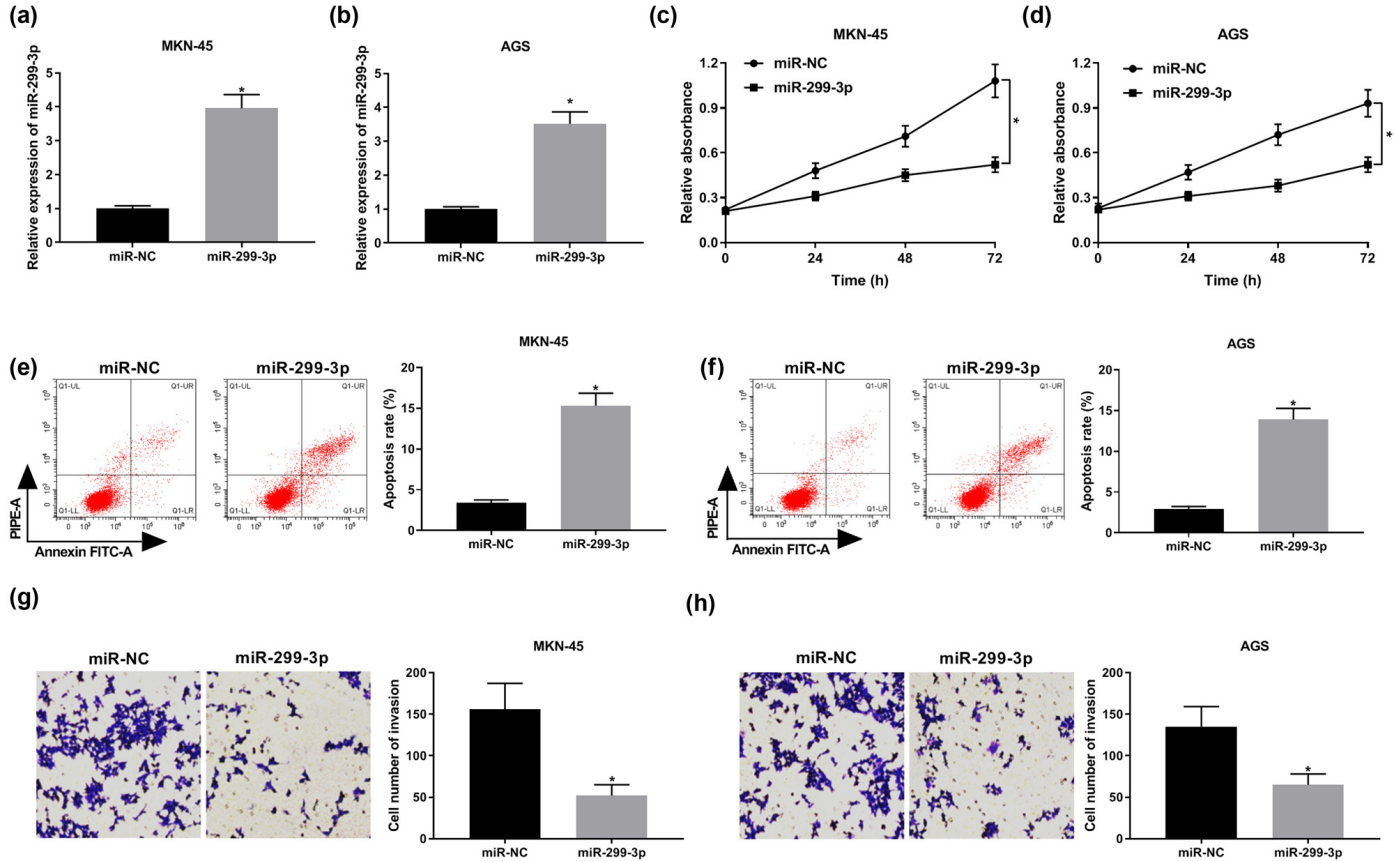
miR-299-3p inhibited cell proliferation and invasion and enhanced apoptosis in GC. (a and b) The expression of miR-299-3p in MKN-45 (a) and AGS cells (b) transfected with miR-299-3p and miR-NC. (c and d) Cell viability of MKN-45 (c) and AGS cells (d) transfected with miR-299-3p and miR-NC for 24, 48, and 72 h detected by CCK8 assay. (e and f) The apoptotic rate of MKN-45 (e) and AGS cells (f) 48 h post-transfection evaluated by flow cytometry. (g and h) Transwell assay was utilized to assess cell invasion ability of MKN-45 (g) and AGS cells (h) 48 h post-transfection. *P < 0.05.

Evaluation of the regulatory effect of miR-299-3p on GC cell proliferation, invasion, and apoptosis was carried out by qRT-PCR, CCK8, flow cytometry, and transwell assay, respectively. The expression of miR-299-3p was elevated significantly in MKN-45 and AGS cells transfected with miR-299-3p mimics compared with the miR-NC group, indicating that the transfection efficiency was extremely high (Figure 2a and b). CCK8 results revealed that the abundance of miR-299-3p obviously hindered cell proliferation ability (Figure 2c and d). Meanwhile, we noticed that the apoptotic rate was enhanced in MKN-45 and AGS cells transfected with miR-299-3p mimics compared with the miR-NC group (Figure 2e and f). Moreover, upregulation of miR-299-3p suppressed cell invasion in GC (Figure 2g and h). Collectively, these results shown that miR-299-3p suppresses proliferation and invasion and facilitates apoptosis in GC.

### 3.3 PAX3 is a target of miR-299-3p

**Figure 3:**
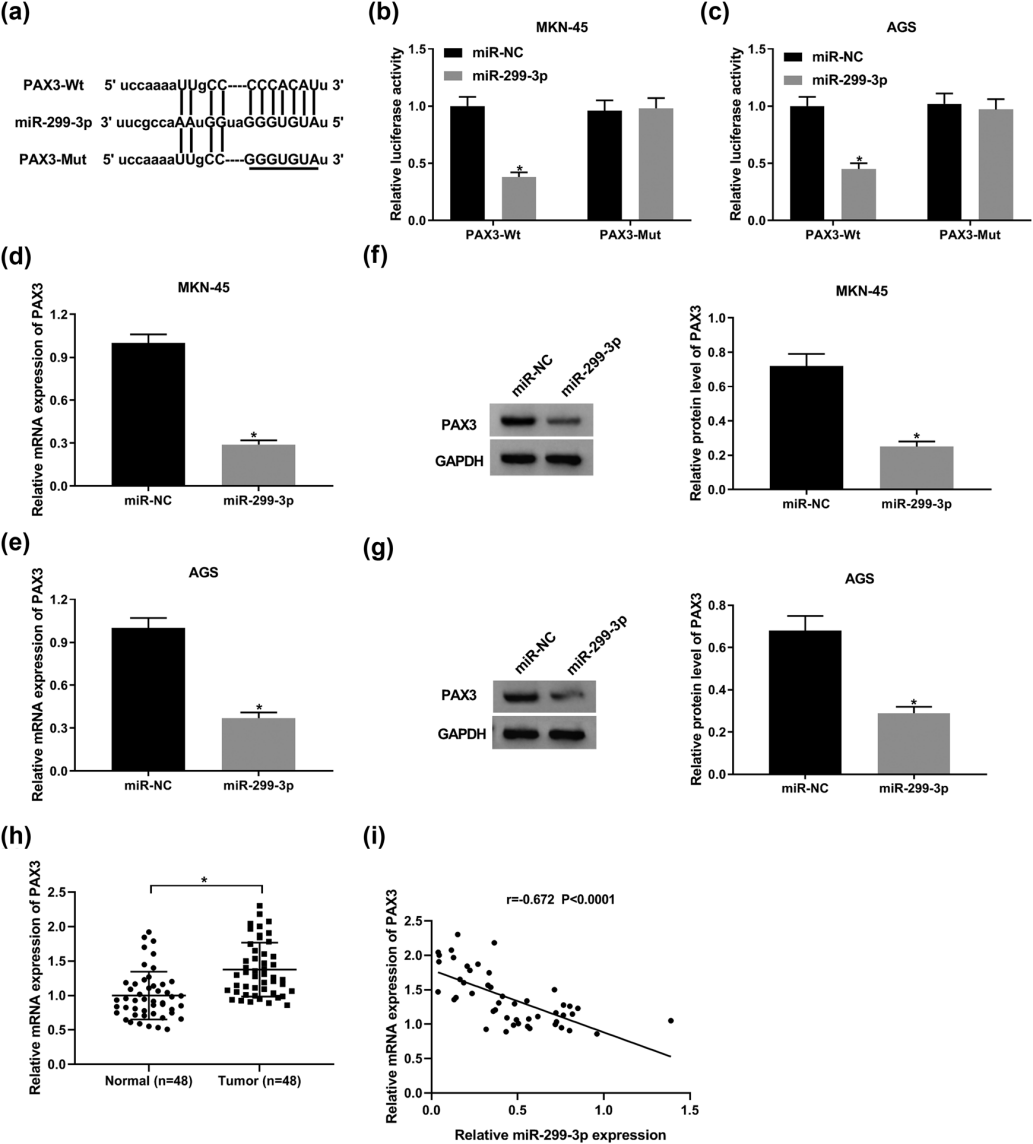
PAX3 is a downstream target of miR-299-3p in GC. (a) Bioinformatics tool StarBase predicted that PAX3 3′-UTR had the binding sites of miR-299-3p. (b and c) Luciferase activity of MKN-45 (b) and AGS cells (c) co-transfected with PAX3-Wt or PAX3-Mut and miR-299-3p or miR-NC. (d and e) The expression of PAX3 mRNA in MKN-45 (d) and AGS cells (e) transfected with miR-299-3p and miR-NC. (f and g) The expression of PAX3 protein in MKN-45 (f) and AGS cells (g) transfected with miR-299-3p and miR-NC. (h) The expression level of PAX3 was detected by qRT-PCR. (i) The correlation between miR-299-3p and PAX3 levels was measured. *P < 0.05.

**Figure A1:**
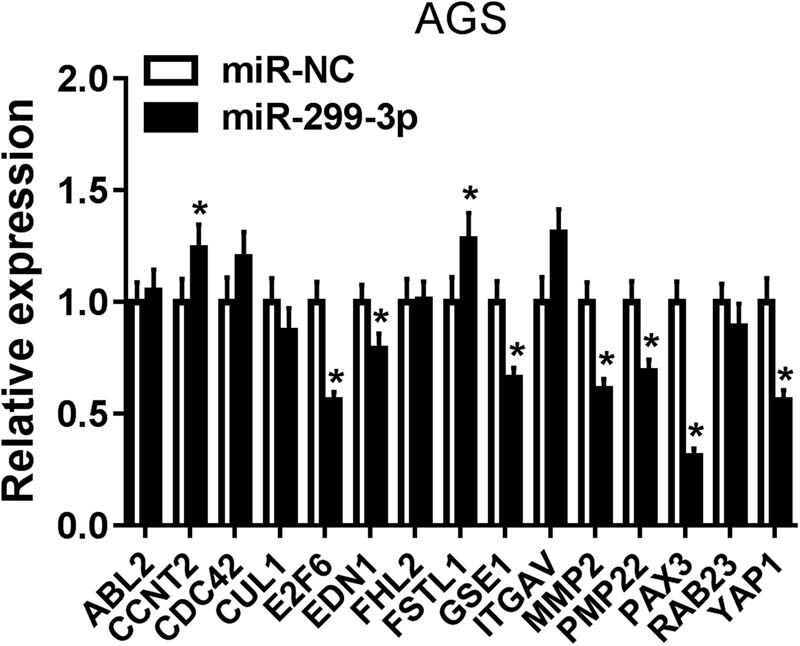
The expression levels of target genes of miR-299-3p were measured by qRT-PCR in AGS cell line.

We used online software, StarBase, miRmap, and miRanda, to computationally search target genes on miR-299-3p. Considering that miRNAs bind their target mRNAs via partial base-pairing within the RNA-Induced Silencing Complexes (RISC), and in consequence, promote translational suppression and/or RNA degradation, the predicted genes with GC promotion effect were further screened out. qRT-PCR was conducted to detect the effects of miR-299-3p on the 15 candidate genes’ expression. As shown in Figure A1, PAX3 is remarkably downregulated when miR-299-3p was upregulated in GC. Based on bioinformatics prediction by online database StarBase, we found that miR-299-3p could bind to PAX3 3′-UTR (Figure 3a). To validate that, we have constructed wild-type PAX3 (PAX3-Wt) and mutant-type PAX3 (PAX3-Mut) vectors, which then were co-transfected with miR-299-3p or miR-NC in MKN-45 and AGS cells to establish a luciferase reporter system. As illustrated in Figure 3b and c, miR-299-3p reduced the fluorescence activity of PAX3-Wt. However, the fluorescence activity of PAX3-Mut was unchanged after miR-299-3p transfection. Moreover, the expression of PAX3 mRNA was decreased in MKN-45 and AGS cells transfected with miR-299-3p (Figure 3d and e). Similarly, upregulation of miR-299-3p repressed PAX3 protein expression (Figure 3f and g). Furthermore, PAX3 was upregulated in tumor tissues compared with normal tissues (Figure 3h), and there was a reverse correlation between miR-299-3p and PAX3 expression levels in GC tissues (Figure 3i). All the data indicated that PAX3 is a downstream target of miR-299-3p in GC.

### 3.4 PAX3 depletion inhibits cell progression and induces apoptosis in GC

**Figure 4:**
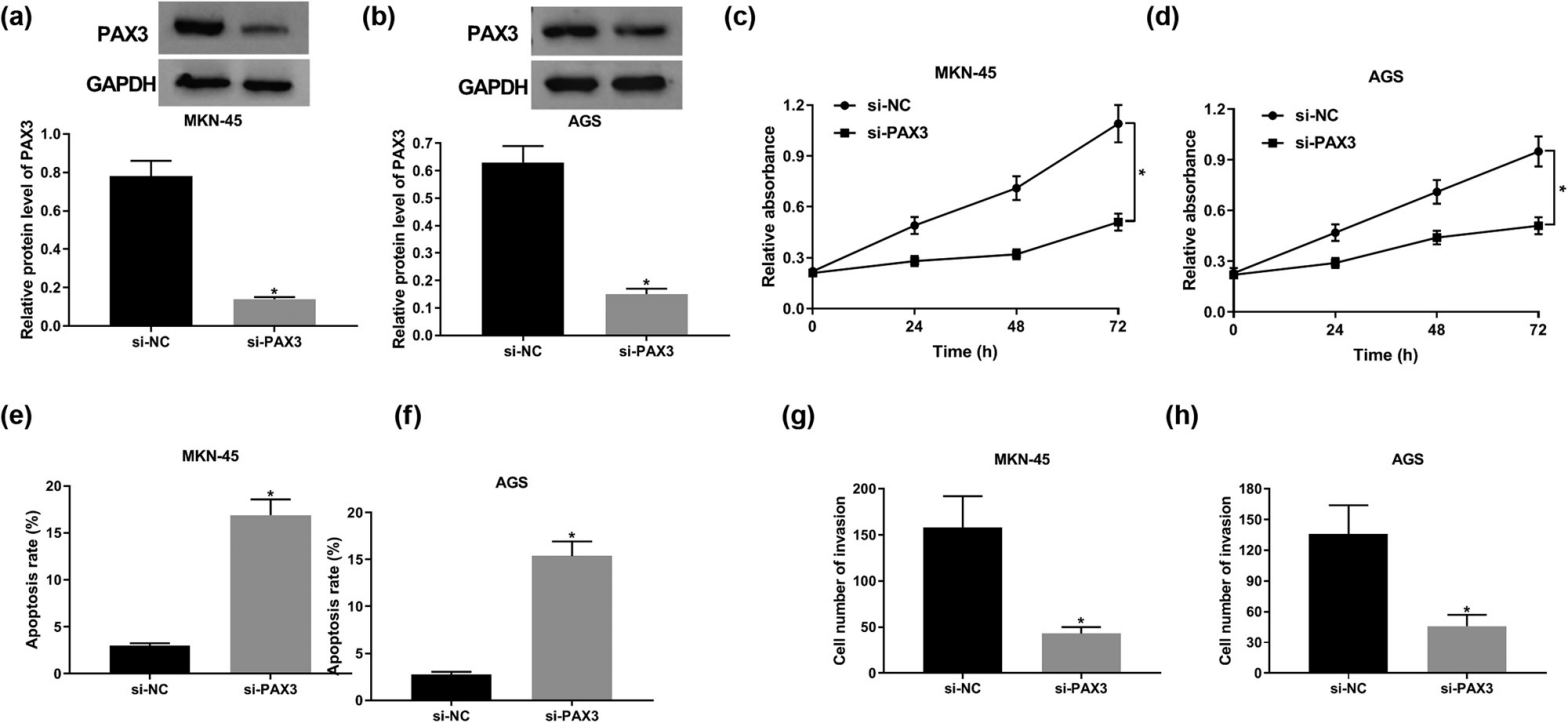
PAX3 knockdown repressed proliferation and invasion and induced apoptosis in GC. (a and b) The expression of PAX3 protein in MKN-45 (a) and AGS cells (b) transfected with si-PAX3 and si-NC. (c and d) Cell viability of MKN-45 (c) and AGS cells (d) transfected with si-PAX3 and si-NC for 24, 48, and 72 h. (e and f) The apoptotic rate of MKN-45 (e) and AGS cells (f) at 48 h post-transfection. (g and h) Cell invasion ability of MKN-45 (g) and AGS cells (h) at 48 h post-transfection. *P < 0.05.

We hypothesized that miR-299-3p exerts its cell regulation function by binding to the target gene PAX3. Thus, we transfected si-PAX3 and si-NC in MKN-45 and AGS cells for the subsequent detection. We observed that PAX3 protein level was significantly lower in MKN-45 and AGS cells after PAX3 knockdown compared with the si-NC group (Figure 4a and b). Moreover, PAX3 silencing significantly attenuated GC cell proliferation at 24, 48, and 72 h post-transfection (Figure 4c and d). Likewise, the number of invasive cells decreased distinctly in si-PAX3 transfection cells compared with si-NC transfection cells (Figure 4g and h). Oppositely, the abundance of PAX3 induced cell apoptosis markedly (Figure 4e and f). Taken together, these results suggest that PAX3 depletion inhibits cell progression and induces apoptosis in GC.

### 3.5 Restoration of PAX3 attenuated miR-299-3p-induced inhibition on GC cell proliferation and invasion

**Figure 5:**
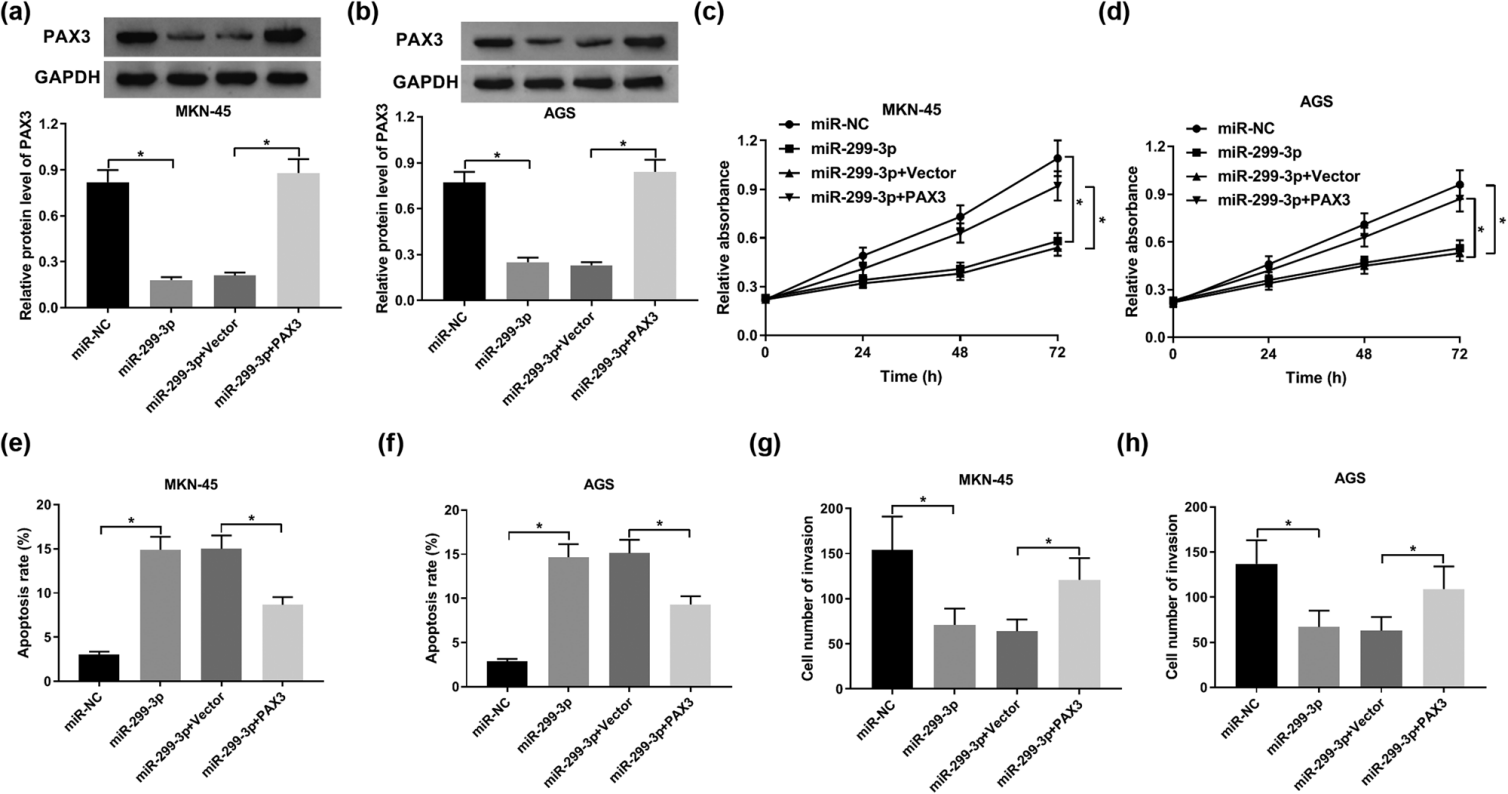
PAX3 abrogated the suppressive effect of miR-299-3p on proliferation and invasion in GC. MKN-45 and AGS cells were transfected with miR-299-3p + PAX3, miR-299-3p + vector, miR-299-3p, and miR-NC. (a and b) The expression of PAX3 protein in MKN-45 (a) and AGS cells (b) at 48 h post-transfection. (c and d) Cell viability of MKN-45 (c) and AGS cells (d) after transfection for 24, 48, and 72 h. (e and f) The apoptotic rate of MKN-45 (e) and AGS cells (f) at 48 h post-transfection. (g and h) Cell invasion ability of MKN-45 (g) and AGS cells (h) at 48 h post-transfection. *P < 0.05.

To explore the regulatory mechanism of miR-299-3p/PAX3 axis in GC cell growth, MKN-45 and AGS cells were transfected with miR-299-3p, miR-299-3p + PAX3, miR-299-3p + vector, and miR-NC. Western blot results exhibited that PAX3 protein was reduced by miR-299-3p, and this effect was reversed by PAX3 plasmid transfection in MKN-45 and AGS cells (Figure 5a and b). Moreover, PAX3 reversed miR-299-3p-induced inhibition on cell proliferation in GC (Figure 5c and d). Besides, low levels of PAX3 accelerated apoptosis, while restoration of PAX3 suppressed apoptosis (Figure 5e and f). Cell invasion ability was inhibited by downregulation of PAX3 expression. However, PAX3 rescued the inhibition of miR-299-3p on cell invasion (Figure 5g and h). These findings clarified that restoration of PAX3 could rescue miR-299-3p-induced inhibition on GC cell proliferation and invasion.

### 3.6 Overexpression of miR-299-3p inhibited the growth of GC *in vivo*

**Figure 6:**
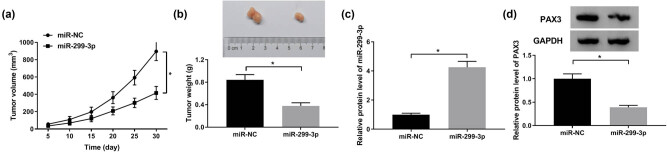
Overexpression of miR-299-3p inhibited the growth of GC *in vivo*. AGS cells stable transfected with miR-NC and miR-299-3p were injected into the hind leg subcutaneous of nude mice (each group of 5) to establish GC xenograft models. (a and b) Tumors’ volume and weight with miR-NC or miR-299-3p were examined. (c) Expression level of miR-299-3p or PAX3 was detected by western blot. *P < 0.05.

To further investigate the efficacy of miR-299-3p in GC, we constructed GC tumor xenograft models. As shown in Figure 6a and b, the tumor volume and weight of mice injected with AGS cells stable transfected with miR-299-3p were significantly decreased compared with those of miR-NC groups. Furthermore, in tumors from mice injected with miR-299-3p overexpressing cells, miR-299-3p expression was remarkably upregulated, while PAX became downregulated (Figure 6c and d).

## 4 Discussion

It is well acknowledged that miRNAs play pivotal roles in the physiological and pathological processes of multiple cancers, such as hepatocellular carcinoma, nasopharyngeal carcinoma, glioma, and GC [26,27,28]. As an oncogene or tumor suppressor, miRNA is involved in tumorigenesis by gene expression modulation at the posttranscriptional level [29,30,31]. Recently, the aberrant expression of miR-299-3p was identified to be associated with malignant clinicopathological characteristics and poor prognosis [32]. For example, miR-299-3p acted as a tumor suppressor, inhibiting cell proliferation and invasion and inducing apoptosis by downregulation of OCT4 or VEGFA expression in renal and colon carcinoma, respectively [33,34]. Conversely, miR-299-3p was upregulated in ovarian cancer tissues and cell lines, and its knockdown reduced proliferation and invasiveness and enhanced apoptosis by targeting OCT4, implicating the oncogenic function of miR-299-3p in ovarian cancer [35]. Thus, the regulatory mechanism of miR-299-3p in GC requires further investigation.

Growing evidence has validated that miRNA exerts its function through interaction with the specific target gene. According to bioinformatics prediction by StarBase, PAX3 is a target gene of miR-299-3p. It is well acknowledged that PAX3 is closely associated with embryonic tissue development, disease formation, and tumorigenesis [36,37,38]. For example, activation of PAX3 controlled muscle precursor cell migration and skeletal muscle formation during forelimb muscle development [39]. PAX3 also contributes to glioblastoma tumorigenesis and differentiation by suppressing p53 transcriptionally [40]. In addition, overexpression of PAX3 was reported to induce cell aggregation and interfere with commissural axon projection *in vitro* and *in vivo* during embryonic spinal cord development [41]. PAX3 is often considered an oncogene, although there are contradicting reports emphasizing the growth-inhibiting potential of PAX3. The ectopic expression of PAX3 dramatically inhibited thyroid cancer progression *in vitro* and *in vivo* through inhibiting the activity of PI3K/Akt and MAPK signaling pathways and promoting the expression and activity of transcription factor FOXO3 [42]. Overexpression of miR-29 and 206 downregulates cell cycle gene expression and induces cell cycle arrest through stabilization of PAX3 in rhabdomyosarcoma, suggesting a tumor suppressor role for PAX3 [43]. Whether miR-299-3p modulates cell progression by targeting PAX3 is still unclear.

In this study, we have assumed that miR-299-3p acts as a tumor suppressor during GC cell growth by base-pairing the target gene PAX3. To confirm this, we have measured the miR-299-3p level in GC tumor tissues and cell lines and discovered that it was downregulated in tumors. More importantly, the abundance of miR-299-3p repressed cell proliferation and invasion and induced apoptosis, implicating that miR-299-3p acts as a tumor suppressor in GC. The subsequent luciferase reporter assay further validated the interaction between miR-299-3p and PAX3. The evaluation of PAX3 mRNA and protein in miR-299-3p transfection cells revealed that PAX3 was modulated by miR-299-3p. PAX3 silencing retarded proliferation and invasion, while enhanced apoptosis in MKN-45 and AGS cells. In addition, recovery of PAX3 expression abolished the suppressive effect mediated by miR-299-3p on GC cell progression.

**Figure 7:**
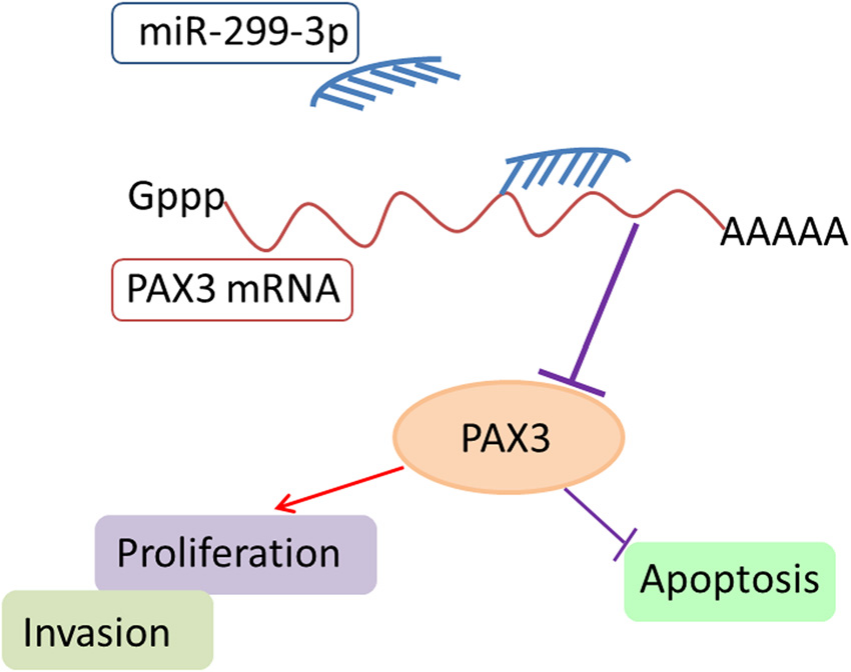
miR-299-3p induced apoptosis and inhibited proliferation and invasion through regulating PAX3.

In conclusion, we clarified the biological mechanism of the miR-299-3p/PAX3 axis on GC cell proliferation, invasion, and apoptosis (Figure 7). We found that miR-299-3p was closely associated with GC cell progression, and depletion of miR-299-3p reduced cell progression and enhanced apoptosis in GC. Moreover, the results illustrated that miR-299-3p suppressed proliferation and invasion by targeting PAX3, representing the potential biomarkers for novel targeted drug development.

## References

[1] Badr El-Din NK, Abdel Fattah SM, Pan D, Tolentino L, Ghoneum M. Chemopreventive activity of MGN-3/biobran against chemical induction of glandular stomach carcinogenesis in rats and its apoptotic effect in gastric cancer cells. Integr Cancer Ther. 2016;15:NP26–34. 10.1177/1534735416642287 PMC5739167 27151588

[2] Yamaguchi T, Fushida S, Yamamoto Y, Tsukada T, Kinoshita J, Oyama K, et al. Tumor-associated macrophages of the M2 phenotype contribute to progression in gastric cancer with peritoneal dissemination. Gastric Cancer. 2016;19:1052–65. 10.1007/s10120-015-0579-8 PMC5034006 26621525

[3] Kiso M, Yoshihara M, Ito M, Inoue K, Kato K, Nakajima S, et al. Characteristics of gastric cancer in negative test of serum anti-helicobacter pylori antibody and pepsinogen test: a multicenter study. Gastric Cancer. 2017;20:764–71. 10.1007/s10120-016-0682-5 28025702

[4] Miao Z, Guo X, Tian L. The long non-coding RNA NORAD promotes the growth of gastric cancer cells by sponging miR-608. Gene. 2019;687:116–24. 10.1016/j.gene.2018.11.052 30453063

[5] Saito R, Kawaguchi Y, Akaike H, Shiraishi K, Maruyama S, Shimizu H, et al. Prognostic significance of lymph node dissection along the upper-third-stomach in patients with lower-third gastric cancer. Anticancer Res. 2019;39:1485–9. 10.21873/anticanres.13266 30842186

[6] Kim SY, Yoon MJ, Park YI, Kim MJ, Nam BH, Park SR. Nomograms predicting survival of patients with unresectable or metastatic gastric cancer who receive combination cytotoxic chemotherapy as first-line treatment. Gastric Cancer. 2018;21:453–63. 10.1007/s10120-017-0756-z 28828688

[7] Ma C, Luo C, Yin H, Zhang Y, Xiong W, Zhang T, et al. Kallistatin inhibits lymphangiogenesis and lymphatic metastasis of gastric cancer by downregulating VEGF-C expression and secretion. Gastric Cancer. 2018;21:617–31. 10.1007/s10120-017-0787-5 29243194

[8] Wang D, Bao F, Teng Y, Li Q, Li J. MicroRNA-506-3p initiates mesenchymal-to-epithelial transition and suppresses autophagy in osteosarcoma cells by directly targeting SPHK1. Biosci Biotechnol Biochem. 2019;83(5):836–44. 10.1080/09168451.2019.1569496 30669957

[9] Wang Y, Jiaqi C, Zhaoying C, Huimin C. MicroRNA-506-3p regulates neural stem cell proliferation and differentiation through targeting TCF3. Gene. 2016;593:193–200. 10.1016/j.gene.2016.08.026 27538704

[10] Chen DL, Yang KY. Berberine alleviates oxidative stress in islets of diabetic mice by inhibiting miR-106b expression and up-regulating SIRT1. J Cell Biochem. 2017;118:4349–57. 10.1002/jcb.26089 28436095

[11] Tao Y, Wang Z, Wang L, Shi J, Guo X, Zhou W, et al. Downregulation of miR-106b attenuates inflammatory responses and joint damage in collagen-induced arthritis. Rheumatol (Oxf). 2017;56:1804–13. 10.1093/rheumatology/kex233 28957555

[12] Dai Z, Jin Y, Zheng J, Liu K, Zhao J, Zhang S, et al. MiR-217 promotes cell proliferation and osteogenic differentiation of BMSCs by targeting DKK1 in steroid-associated osteonecrosis. Biomed Pharmacother. 2019;109:1112–9. 10.1016/j.biopha.2018.10.166 30551361

[13] Zhu M, Wei C, Lin J, Dong S, Gao D, Chen J, et al. UHRF1 is regulated by miR-124-3p and promotes cell proliferation in intrahepatic cholangiocarcinoma. J Cell Physiol. 2019;234(11):19875–85. 10.1002/jcp.28586 30989656

[14] Wu SQ, Zhang LH, Huang HB, Li YP, Niu WY, Zhan R. miR-299-5p promotes cell growth and regulates G1/S transition by targeting p21Cip1/Waf1 in acute promyelocytic leukemia. Oncol Lett. 2016;12:741–7. 10.3892/ol.2016.4621 PMC4907288 27347210

[15] Chen X, Qi M, Yang Q, Li. JY. MiR-299-3p functions as a tumor suppressor in thyroid cancer by regulating SHOC2. Eur Rev Med Pharmacol Sci. 2019;23:8. 10.26355/eurrev_201901_16769 30657565

[16] Zheng D, Dai Y, Wang S, Xing X. MicroRNA-299-3p promotes the sensibility of lung cancer to doxorubicin through directly targeting ABCE1. Int J Clin Exp Pathol. 2015;8:10072–81. PMC4637529 26617714

[17] Muratovska A, Zhou C, He S, Goodyer P, Eccles MR. Paired-box genes are frequently expressed in cancer and often required for cancer cell survival. Oncogene. 2003;22:7989–97. 10.1038/sj.onc.1206766 12970747

[18] Lin S, Ren A, Wang L, Santos C, Huang Y, Jin L, et al. Aberrant methylation of Pax3 gene and neural tube defects in association with exposure to polycyclic aromatic hydrocarbons. Clin Epigenet. 2019;11:13. 10.1186/s13148-019-0611-7 PMC6341549 30665459

[19] Cao S, Du J, Lv Y, Lin H, Mao Z, Xu M, et al. PAX3 inhibits beta-Tubulin-III expression and neuronal differentiation of neural stem cell. Biochem Biophys Res Commun. 2017;485:307–11. 10.1016/j.bbrc.2017.02.086 28223217

[20] Boudjadi S, Chatterjee B, Sun W, Vemu P, Barr FG. The expression and function of PAX3 in development and disease. Gene. 2018;666:145–57. 10.1016/j.gene.2018.04.087 PMC6624083 29730428

[21] Liang X, Dong Z, Bin W, Dekang N, Xuhang Z, Shuyuan Z, et al. PAX3 promotes proliferation of human glioma cells by WNT/beta-catenin signaling pathways. J Mol Neurosci. 2019;68:66–77. 10.1007/s12031-019-01283-2 30826985

[22] Akaike K, Suehara Y, Kohsaka S, Hayashi T, Tanabe Y, Kazuno S, et al. PPP2R1A regulated by PAX3/FOXO1 fusion contributes to the acquisition of aggressive behavior in PAX3/FOXO1-positive alveolar rhabdomyosarcoma. Oncotarget. 2018;9:9. 10.18632/oncotarget.25392 PMC5982774 29861864

[23] Liu W, Sui F, Liu J, Wang M, Tian S, Ji M, et al. PAX3 is a novel tumor suppressor by regulating the activities of major signaling pathways and transcription factor FOXO3a in thyroid cancer. Oncotarget. 2016;7(34):54744–57. 10.18632/oncotarget.10753 PMC5342378 27458157

[24] Zhang L, Xia L, Zhao L, Chen Z, Shang X, Xin J, et al. Activation of PAX3-MET pathways due to miR-206 loss promotes gastric cancer metastasis. Carcinogenesis. 2015;36:390–9. 10.1093/carcin/bgv009 25653235

[25] Yan C, Zhu M, Ding Y, Yang M, Wang M, Li G, et al. Meta-analysis of genome-wide association studies and functional assays decipher susceptibility genes for gastric cancer in Chinese populations. Gut. 2020;69:641–51. 10.1136/gutjnl-2019-318760 31383772

[26] Lin C, Zong J, Lin W, Wang M, Xu Y, Zhou R, et al. EBV-miR-BART8-3p induces epithelial-mesenchymal transition and promotes metastasis of nasopharyngeal carcinoma cells through activating NF-kappaB and Erk1/2 pathways. J Exp Clin Cancer Res. 2018;37:283. 10.1186/s13046-018-0953-6 PMC6257964 30477559

[27] Huang X, Gao Y, Qin J, Lu S. lncRNA MIAT promotes proliferation and invasion of HCC cells via sponging miR-214. Am J Physiol Gastrointest liver Physiol. 2018;314:G559–65. 10.1152/ajpgi.00242.2017 29097358

[28] Sun D, Mu Y, Piao H. MicroRNA-153-3p enhances cell radiosensitivity by targeting BCL2 in human glioma. Biol Res. 2018;51:56. 10.1186/s40659-018-0203-6 PMC6288870 30537994

[29] Huang S, Zheng S, Huang S, Cheng H, Lin Y, Wen Y, et al. Flot2 targeted by miR-449 acts as a prognostic biomarker in glioma. Artif Cell Nanomed Biotechnol. 2019;47:250–5. 10.1080/21691401.2018.1549062 30663389

[30] Chen J, Wu Z, Zhang Y. LncRNA SNHG3 promotes cell growth by sponging miR-196a-5p and indicates the poor survival in osteosarcoma. Int J Immunopathol Pharmacol. 2019;33:2058738418820743. 10.1177/2058738418820743 PMC6329016 30791797

[31] Lai YY, Shen F, Cai WS, Chen JW, Feng JH, Cao J, et al. MiR-384 regulated IRS1 expression and suppressed cell proliferation of human hepatocellular carcinoma. Tumour Biol. 2016;37:14165–71. 10.1007/s13277-016-5233-5 27542674

[32] Dang S, Zhou J, Wang Z, Wang K, Dai S, He S. MiR-299-3p functions as a tumor suppressor via targeting Sirtuin 5 in hepatocellular carcinoma. Biomed Pharmacother. 2018;106:966–75. 10.1016/j.biopha.2018.06.042 30170358

[33] Gohring AR, Reuter S, Clement JH, Cheng X, Theobald J, Wolfl S, et al. Human microRNA-299-3p decreases invasive behavior of cancer cells by downregulation of Oct4 expression and causes apoptosis. PLoS One. 2017;12:e0174912. 10.1371/journal.pone.0174912 PMC5398498 28426762

[34] Wang JY, Jiang JB, Li Y, Wang YL, Dai Y. MicroRNA-299-3p suppresses proliferation and invasion by targeting VEGFA in human colon carcinoma. Biomed Pharmacother. 2017;93:1047–54. 10.1016/j.biopha.2017.07.030 28738498

[35] Zhao R, Liu Q, Lou C. MicroRNA-299-3p regulates proliferation, migration and invasion of human ovarian cancer cells by modulating the expression of OCT4. Arch Biochem Biophys. 2018;651:21–7. 10.1016/j.abb.2018.05.007 29758200

[36] Der Vartanian A, Quetin M, Michineau S, Aurade F, Hayashi S, Dubois C, et al. PAX3 confers functional heterogeneity in skeletal muscle stem cell responses to environmental stress. Cell Stem Cell. 2019;24(6):958e9. 10.1016/j.stem.2019.03.019 PMC6628901 31006622

[37] Xu M, Li Y, Du J, Lin H, Cao S, Mao Z, et al. PAX3 promotes cell migration and CXCR4 gene expression in neural crest cells. J Mol Neurosci. 2018;64:1–8. 10.1007/s12031-017-0995-9 29134414

[38] Wei C, Ren L, Li K, Lu Z. The regulation of survival and differentiation of neural stem cells by miR-124 via modulating PAX3. Neurosci Lett. 2018;683:19–26. 10.1016/j.neulet.2018.05.051 29864453

[39] Shin J, Watanabe S, Hoelper S, Kruger M, Kostin S, Poling J, et al. BRAF activates PAX3 to control muscle precursor cell migration during forelimb muscle development. Elife. 2016;5:e18351. 10.7554/eLife.18351 PMC5148607 27906130

[40] Zhu H, Wang H, Huang Q, Liu Q, Guo Y, Lu J, et al. Transcriptional repression of p53 by PAX3 contributes to gliomagenesis and differentiation of glioma stem cells. Front Mol Neurosci. 2018;11:187. 10.3389/fnmol.2018.00187 PMC6003214 29937714

[41] Lin J, Fu S, Yang C, Redies C. Pax3 overexpression induces cell aggregation and perturbs commissural axon projection during embryonic spinal cord development. J Comp Neurol. 2017;525:1618–32. 10.1002/cne.24146 27864937

[42] Liu W, Sui F, Liu J, Wang M, Tian S, Ji M, et al. PAX3 is a novel tumor suppressor by regulating the activities of major signaling pathways and transcription factor FOXO3a in thyroid cancer. Oncotarget. 2016;7:54744–57. 10.18632/oncotarget.10753 PMC5342378 27458157

[43] Li L, Sarver AL, Alamgir S, Subramanian S. Downregulation of microRNAs miR-1, -206 and -29 stabilizes PAX3 and CCND2 expression in rhabdomyosarcoma. Lab Invest J Tech Methods Pathol. 2012;92:571–83. 10.1038/labinvest.2012.10 22330340

